# Optimising Soft-Tissue Balancing in Hip Hemiarthroplasty Surgery Using a Simple Planning Protocol

**DOI:** 10.7759/cureus.50280

**Published:** 2023-12-10

**Authors:** Narada R Karuna Pathirannehelage, Lamindu Niroshana, Manoj Sood

**Affiliations:** 1 Trauma and Orthopaedics, Bedfordshire Hospitals NHS Foundation Trust, Bedford, GBR

**Keywords:** preoperative templating, scaled radiograph, soft tissue balancing, leg length, horizontal offset, hip hemiarthroplasty, intracapsular fracture

## Abstract

Introduction

Intracapsular neck of the femur fractures are some of the most common fragility fractures with significant morbidity and mortality. Cemented hemiarthroplasty is the standard treatment in most cases. Restoring the horizontal offset and leg length is important to optimize the outcome of hip hemiarthroplasty. Preoperative templating based on a scaled radiograph is common prior to total hip arthroplasty surgery to achieve optimum offset and leg length. It is not routine to have scaled radiographs available prior to a hemiarthroplasty surgery. Our simple non-scaled radiograph templating protocol (NSRTP) was introduced to help establish the correct offset and leg length in the absence of scaled radiographs.

Methods

A retrospective, comparative, case-control study was carried out in an acute hospital setting. Scaled radiographs were not available for any patients in the study, as is usual for hemiarthroplasty patients in our hospital. One group had surgery without any templating. The other group had surgery using the NSRTP. The NSRTP determined optimal ipsilateral offset based on preoperative measurement of the contralateral hip offset and ipsilateral head diameter on unscaled radiographs together with intraoperative measurement of the diameter of the ipsilateral femoral head removed at surgery. To help achieve the correct length, the NSRTP also included assessment and restoration of the contralateral greater trochanter tip-to-head relationship. The neck cut was tailored to restore the correct relationship.

Results

Twenty-three patients underwent hemiarthroplasty surgery without any templating and 23 had surgery using the NSRTP. The implants used were C-STEM™ (DePuy Synthes, Raynham, Massachusetts, United States) and SPECTRON (Smith & Nephew plc, London, United Kingdom); stems were used together with monopolar heads. The stems were available in standard and high offset versions and with a variety of neck lengths, allowing the correct combination to be selected to restore offset. When the NSRTP was used, horizontal offset and leg length were restored to within 2 mm of the contralateral hip in 22 patients out of 23. There was a statistically significant improvement in restoration of offset and leg length when the NSRTP was used, compared to the control group.

Conclusion

Restoration of the offset and leg length is important to maximize the outcome of hip arthroplasty surgery. Preoperative templating is helpful to achieve offset and leg length in total hip replacement. In the absence of scaled radiographs, NSRTP enables restoration of offset and leg length to within 2 mm of normal in more than 96% of patients. This protocol requires knowledge of the offset of the hemiarthroplasty stems being used, which is easily available from the relevant manufacturer.

## Introduction

Intracapsular neck of the femur fractures are some of the most common fragility fractures. They comprise half of all proximal femoral fractures and are one of the most important causes of functional failure, morbidity, and mortality in elderly patients [1-3]. Due to an increasing life expectancy, the incidence of these fractures is expected to increase over the years. The standard treatment for intracapsular neck of the femur fractures is hip arthroplasty and in a majority of cases, hemiarthroplasty is performed. Recent updates to the National Institute for Health and Care Excellence (NICE) guidelines have stated that hemiarthroplasty rather than total hip replacement (THR) is recommended for most patients [4].

Minimising complications in hemiarthroplasty is important as many of these patients have low reserve and deteriorate rapidly in the presence of complications. The stability of the hip is important to prevent hip dislocation and to help to optimize rehabilitation. Restoring horizontal hip offset and leg length is important to maintain hip stability and improve function [5-11]. Preoperative templating using scaled radiographs is standard practice for THRs and this helps to restore the hip offset and leg length.

Current literature does not provide a method to allow templating to optimize hip offset and leg length in the absence of scaled radiographs. Our proposed simple non-scaled radiograph templating protocol (NSRTP) was developed to provide a solution to allow optimization of hip offset and leg length in the absence of scaled radiographs.

## Materials and methods

A retrospective, comparative study was carried out in Bedford Hospital, Bedfordshire Hospitals NHS Foundation Trust, Bedford, United Kingdom. All patients who underwent unipolar modular hemiarthroplasty for intracapsular neck of the femur fracture between January 1, 2022, and June 30, 2022, were included. Patients with previous contralateral hip procedures (fixation, arthroplasty, osteotomy, etc.) were excluded as our method relies on measurements from the other hip.

Our NSRTP was used in patients who underwent unipolar modular hemiarthroplasty under the care of the senior author (MS). These patients were compared with patients who underwent the same procedure but without any templating (the usual standard of care) during the same period. C-STEM™ (DePuy Synthes, Raynham, Massachusetts, United States) and SPECTRON (Smith & Nephew plc, London, United Kingdom) stems were used in all cases. The stems were available in standard and high offset versions and with a variety of neck lengths, allowing the correct combination to be selected to restore offset. Patients who underwent unipolar modular hemiarthroplasty without any templating are termed Group A and patients who underwent unipolar modular hemiarthroplasty using the NSRTP are considered Group B.

NSRTP

Horizontal Offset

**Figure 1:**
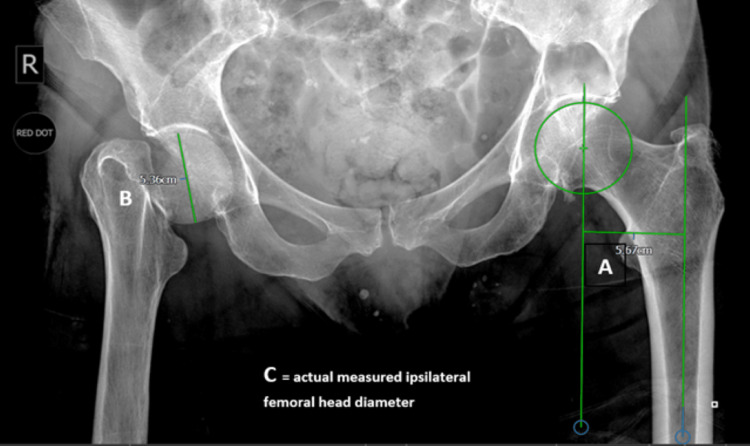
Preoperative radiograph with measurements

The ipsilateral (fracture side) head diameter (unscaled femoral head diameter) measured from the preoperative radiograph was used together with the actual intraoperative head diameter of the retrieved femoral head to scale the preoperative radiograph. This allowed the calculation of the actual offset from the contralateral offset measured on the preoperative radiograph (unscaled offset) (Figure 1, Table 1). The calculation was done as follows: Target of set calculation = (Actual measured femoral head diameter C ÷ Unscaled ipsilatera head diameter B) X unscaled contralateral hip offset A. This was the target offset to be reproduced by selection of a standard or high offset stem together with the femoral head with correct taper length with reference to the manufacturers' offset chart for the femoral prostheses being used (Table 2). Trialling was undertaken before definitive implantation.

**Table 1:** An example of offset calculation

Measurement	Value
Unscaled contralateral offset measurement: A	56.7
Unscaled ipsilateral femoral head diameter: B	53.6
Actual intraoperative measured ipsilateral femoral head diameter: C	45
Actual offset (C/B*A)	47.6

**Table 2:** Offset chart for SPECTRON* implants *Smith & Nephew plc, London, United Kingdom

Horizontal offset in mm	Taper size				
Stem size	-3	0	+4	+8	+12
1	32	35	38	41	44
1H	38	41	44	47	50
2	34	36	39	42	45
2H	42	44	47	50	53
3	35	38	41	44	47
3H	45	48	51	54	57
4	37	39	42	45	48
4H	47	49	52	55	58

In the case above, if a size 2 SPECTRON stem is the correct size, a high offset version with a +4 head will provide an offset of 47, which is close to the target offset of 47.6.

Leg Length

**Figure 2:**
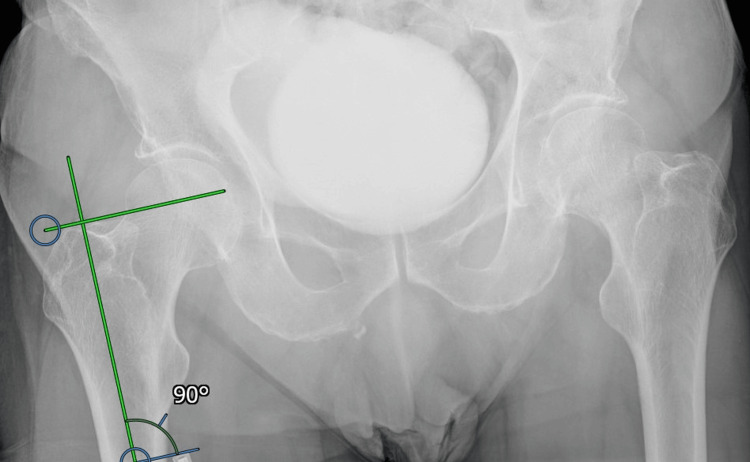
Assessment of preoperative greater trochanter to head relationship

**Figure 3:**
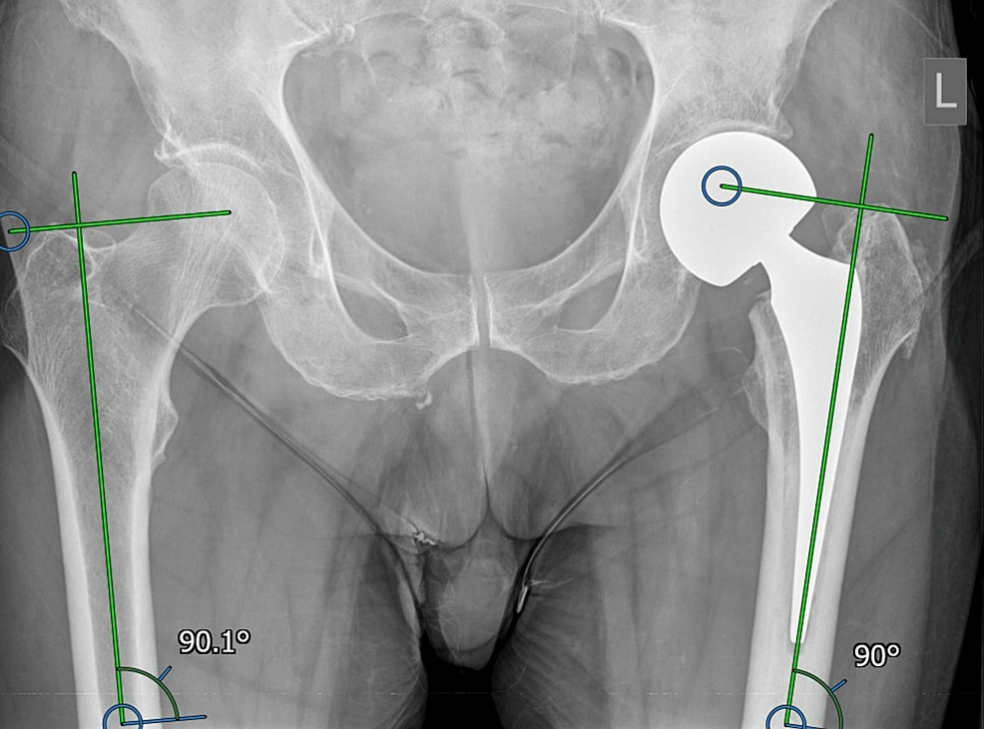
Assessment of postoperative greater trochanter to head relationship

The relationship of the tip of the greater trochanter to the femoral head on the contralateral side was determined (Figure 2) and we aimed to reproduce this relationship on the fractured side with the hemiarthroplasty (Figure 3)

The offset and leg length were measured digitally on postoperative radiographs to determine how accurately these parameters had been reproduced. Leg length was measured only at the level of the hips on pelvic radiographs as this was what the study was investigating.

The difference between the horizontal offset of the implanted prosthesis and the calculated target value was assessed for both groups. The difference between the offset of the two sides on the postoperative radiographs was also assessed. The leg length on the postoperative radiographs was also measured to test how accurately it had been restored.

IBM SPSS Statistics for Windows, Version 26.0 (Released 2019; IBM Corp., Armonk, United States) was used for statistical analysis.

## Results

Twenty-eight patients underwent hemiarthroplasty without any templating (Group A). Of these, five patients were excluded as they had undergone contralateral hip procedures and 23 patients were included in the analysis. Twenty-three patients underwent hemiarthroplasty using the NSRTP (Group B). Thus, both groups had 23 participants each.

Horizontal offset

The average difference in horizontal offset between the implanted prostheses and the calculated value in group A where no templating was performed was −4.16mm and the average difference of horizontal offset between the implanted prostheses and the calculated value in group B where the NSRTP was used was 0.16 mm. An independent t test was used to assess the significance of the difference between the mean of the two populations. For the independent t test, the square of the difference of the offset between the implanted prostheses and the calculated value was used (Table 3) to prevent the effects of plus and minus values from neutralizing each other. Levene’s test for assessment for equality of variance was used. The p-value for Levene’s test is 0.000 and equality of variance was not assumed. The p-value was 0.003 (<0.05) for the independent t test (Table 4), which confirmed a statistically significant difference between the two groups.

**Table 3:** Squared difference of offset between implanted prosthesis and calculated value Group A: patients who underwent unipolar modular hemiarthroplasty without any templating; Group B: patients who underwent unipolar modular hemiarthroplasty using the NSRTP NSRTP: non-scaled radiograph templating protocol

	Group	N	Mean	SD	Standard Error Mean
Squared Difference (Implanted -Calculated)	Group A	23	43.06	59.971	12.505
	Group B	23	1.41	1.549	.323

**Table 4:** Independent t test: squared, offset difference between implanted prosthesis and calculated value

		Levene's test for equality of variances		t-test for Equality of Means						
		F	Sig.	t	df	Sig. (2-tailed)	Mean Difference	Std. Error Difference	95%CI of the difference	
									Lower	Upper
Squared Difference (Implanted -Calculated)	Equal variances assumed	16.202	0.000	3.330	44	0.002	41.651	12.509	16.441	66.862
	Equal variances not assumed			3.330	22.029	0.003	41.651	12.509	15.711	67.591

The difference between the offset of two sides in the postoperative radiographs in group A is −4.35 mm and the difference between the offset of two sides in the postoperative radiographs in group B is 0.22mm. Independent t test used to test the difference between the mean of the two populations. For the independent t test, square of the postoperative difference of the offset between the two sides (Table 5) was used to prevent the effects of plus and minus values neutralizing each other. Levene’s test for assessment for equality of variance was used. The p-value for Levene’s test was 0.000 and equality of variance was not assumed. P-value was 0.001 (<0.05) in the independent t test (Table 6), which confirms the statistically significant difference between the two groups.

**Table 5:** Squared postoperative offset difference between the two sides Group A: patients who underwent unipolar modular hemiarthroplasty without any templating; Group B: patients who underwent unipolar modular hemiarthroplasty using the NSRTP NSRTP: non-scaled radiograph templating protocol

	Group	N	Mean	SD	Standard Error Mean
Squared postoperative offset difference	Group A	23	42.96	51.938	10.830
	Group B	23	1.52	1.563	.326

**Table 6:** Independent t test for two groups: Squared, postoperative offset difference between the two groups

		Levene's test for equality of variances		t-test for equality of means						
		F	Sig.	t	df	Sig. (2-tailed)	Mean Difference	Standard Error Difference	95%CI of the Difference	
									Lower	Upper
Squared Difference (postoperative offset difference)	Equal variances assumed	22.875	0.000	3.824	44	0.000	41.435	10.835	19.599	63.271
	Equal variances not assumed			3.824	22.040	0.001	41.435	10.835	18.967	63.902

In Group A, the offset was restored to within 2 mm of the contralateral side in only five out of 23 patients (range 15 mm to 5 mm). In Group B, the offset was restored within 2 mm of the contralateral side in 22 of the 23 patients (range −2 mm to 3 mm)

Leg length

The difference between the leg length in Group A is −0.34mm and Group B is 0.30 mm. Independent t test was used to test the difference between the mean of the two populations. For independent t test, square of the leg length difference (Table 7) was used for statistical analysis to prevent the effects of plus and minus values from neutralizing each other. Levene’s test for assessment for equality of variance was used. The p-value for Levene’s test was 0.000 and equality of variance was not assumed. P-value was 0.005 (<0.05) in the independent t test (Table 8), which confirms a statistically significant difference between the two groups.

**Table 7:** Squared leg length discrepancy at hip level Group A: patients who underwent unipolar modular hemiarthroplasty without any templating; Group B: patients who underwent unipolar modular hemiarthroplasty using the NSRTP NSRTP: non-scaled radiograph templating protocol

	Group	N	Mean	SD	Standard Error Mean
Leg length discrepancy	Group A	23	28.35	41.635	8.682
	Group B	23	1.26	1.544	.322

**Table 8:** Independent t test for the two groups: squared leg length discrepancy at hip level

		Levene's test for equality of variances		t-test for equality of means						
		F	Sig.	t	df	Sig. (2-tailed)	Mean Difference	Standard Error Difference	95%CI of the Difference	
									Lower	Upper
LLD	Equal variances assumed	26.812	0.000	3.118	44	0.003	27.087	8.688	9.578	44.596
	Equal variances not assumed			3.118	22.060	0.005	27.087	8.688	9.073	45.101

In Group A, leg length was restored within 2 mm of the contralateral side in only nine out of 23 (range −12 mm to 5mm). In Group B, leg length was restored to within 2 mm of the contralateral side in 22 out of 23 patients (−3 mm to 2mm).

## Discussion

Restoration of the horizontal offset and leg length is important to improve the stability and functional outcome of total hip arthroplasty operations [5-11]. Evidence about the importance of restoration of horizontal offset and leg length in hemiarthroplasty in the literature is mixed. There are studies that report poor outcomes when the offset and leg length are not restored [12-15], but other studies did not find any significant relationship between functional outcome and restoration of offset and leg length [16-18]. Considering the biomechanics of the hip joint, achieving the native offset and leg length should logically maximize the function of the hip hemiarthroplasty and help optimize gait and so it seems prudent to take all necessary measures to restore offset and leg length to be as normal as possible. Leg length is not restored in hemiarthroplasty surgery most of the time [19]. The relationship between the tip of the greater trochanter to the femoral head was previously described as a landmark in hemiarthroplasty [20,21].

Our study reports the successful use of an NSRTP to help accurately restore hip offset and leg length when scaled radiographs are not available for preoperative templating. Our NSRTP helped to restore offset and leg length to within 2 mm of the contralateral side in more than 96% of cases. There was a statistically significant difference between the study groups. The standard error of the mean was lower in the group where the NSRTP was used, confirming its superior results.

The only resource required to use the NSRTP is knowledge of the stem offsets of the standard and high offset stem versions and with a variety of neck lengths, allowing the correct combination to be selected to restore offset. This information can be easily found out from the relevant implant company. The same technique could also be used if total hip arthroplasty surgery is performed for intracapsular hip fractures when a scaled radiograph is not available.

There are a number of study limitations. Good quality preoperative radiographs are needed, especially with respect to avoidance of too much rotation of the normal side as this is the side used to determine target offset, and this can be reduced if the hip is unduly rotated. Patients with prior contralateral hip surgery were excluded. However, previous hemiarthroplasty or THA would not exclude the use of this technique. In such cases, the prosthetic femoral head could also be used to scale an unscaled radiograph and thus allow conventional templating. Non-arthroplasty contralateral surgery could make application of the NSRTP more difficult, but this would depend on the surgery performed. Postoperative functional outcomes and patient quality of life were not assessed as part of this study. In addition, this was a non-randomized study performed in a single centre. A larger multicenter study could be useful; however, as the technique is simply a way to scale non-scaled radiographs, we feel that it can immediately be applied in hospitals that perform hemiarthroplasty surgery, without further study.

## Conclusions

The NSRTP offers a simple way to scale a non-scaled radiograph to help optimize offset and leg length in hemiarthroplasty surgery (or total hip arthroplasty surgery) for intracapsular femoral neck fractures. This should help to improve surgical outcomes, hip stability, and, ultimately, patient recovery and mobility. While further studies to confirm the reliability of the technique could be performed, it is a simple scaling method and our findings suggest that this technique should be applied routinely as a templating method for hemiarthroplasty or total hip arthroplasty surgery for intracapsular fractures where no scaled radiograph is available.
